# Radiofrequency ablation of the sinuvertebral nerve for patients with discogenic low back pain following lumbar interbody fusion: a case series study

**DOI:** 10.3389/fneur.2025.1539971

**Published:** 2025-04-22

**Authors:** Lijie Wang, Jie Lu, Hongyan Wang, Liangliang He, Zhi Dou, Wenxing Zhao, Song Yang, Dong Liu, Liqiang Yang

**Affiliations:** ^1^Department of Pain Management, Chengdu Second People's Hospital, Chengdu, China; ^2^Department of Pain Management, Xuanwu Hospital, Capital Medical University, Beijing, China

**Keywords:** RFA—radiofrequency ablation, DLBP, SVN, lumbar interbody fusion, OLIF = oblique lateral interbody fusion

## Abstract

**Background:**

This study aimed to investigate the clinical value of radiofrequency ablation (RFA) of the sinuvertebral nerve (SVN) in the treatment of discogenic low back pain (DLBP) following lumbar interbody fusion.

**Methods:**

A total of 12 patients who underwent RFA of the SVN for DLBP after lumbar interbody fusion at the Pain Department of Xuanwu Hospital of Capital Medical University from February 2023 to August 2023 were included in this retrospective study.

**Results:**

In total, 12 patients with DLBP were included. The preoperative visual analog scale (VAS) score was 7.00(6.00, 7.75), while the postoperative VAS score at 1 day, 1 month, and 3 months was 1.00 (1.00, 1.00). This represented a statistically significant improvement compared to the preoperative period (all *p* = 0.002). The preoperative Pittsburgh Sleep Quality Index (PSQI) score was 14.42 ± 1.83, and the postoperative PSQI scores at 1 month and 3 months were 4.75 ± 1.06 and 2.17 ± 1.11, respectively (all *p* < 0.001).

**Conclusion:**

RFA of the SVN provides satisfactory short-term clinical results in patients with DLBP following lumbar interbody fusion. It appears to be an effective treatment for patients with DLBP who have poor outcomes after open lumbar spine surgery.

## Introduction

Discogenic low back pain (DLBP) originates from lumbar disc degeneration, and nerve admissibility stimulation causes chronic low back pain, with or without lower extremity referred pain (1). Nucleus pulposus removal and intervertebral fusion are performed to treat the affected intervertebral discs; however, often only the nucleus pulposus and part of the annulus fibrosus are removed due to spinal stability considerations. The residual sinuvertebral nerve (SVN) in the residual annulus fibrosus could produce abnormal pain stimulation (2, 3). Radiofrequency ablation (RFA) has been widely used in the treatment of various types of neuropathic pain and has demonstrated good efficacy (4–6). It causes degeneration and necrosis of Aδ and C nerve fibers that transmit nociceptive signals through the effects of thermal injury, ultimately relieving pain. Therefore, for patients with DLBP following lumbar interbody fusion who have poor outcomes, further RFA of the SVN could block the source of pain and the pain transduction pathway. This study aimed to evaluate whether radiofrequency ablation of the SVN could reduce pain and improve clinical outcomes in patients with DLBP following lumbar interbody fusion.

## Methods

### Study patients

A total of 12 patients who underwent RFA of the SVN for DLBP following lumbar interbody fusion at the Pain Department of Xuanwu Hospital of Capital Medical University from February 2023 to August 2023 were included in this retrospective study. The Ethics Committee of Xuanwu Hospital of Capital Medical University approved this study (2021117). Informed consent was not required for this retrospective case series.

**Figure 1:**
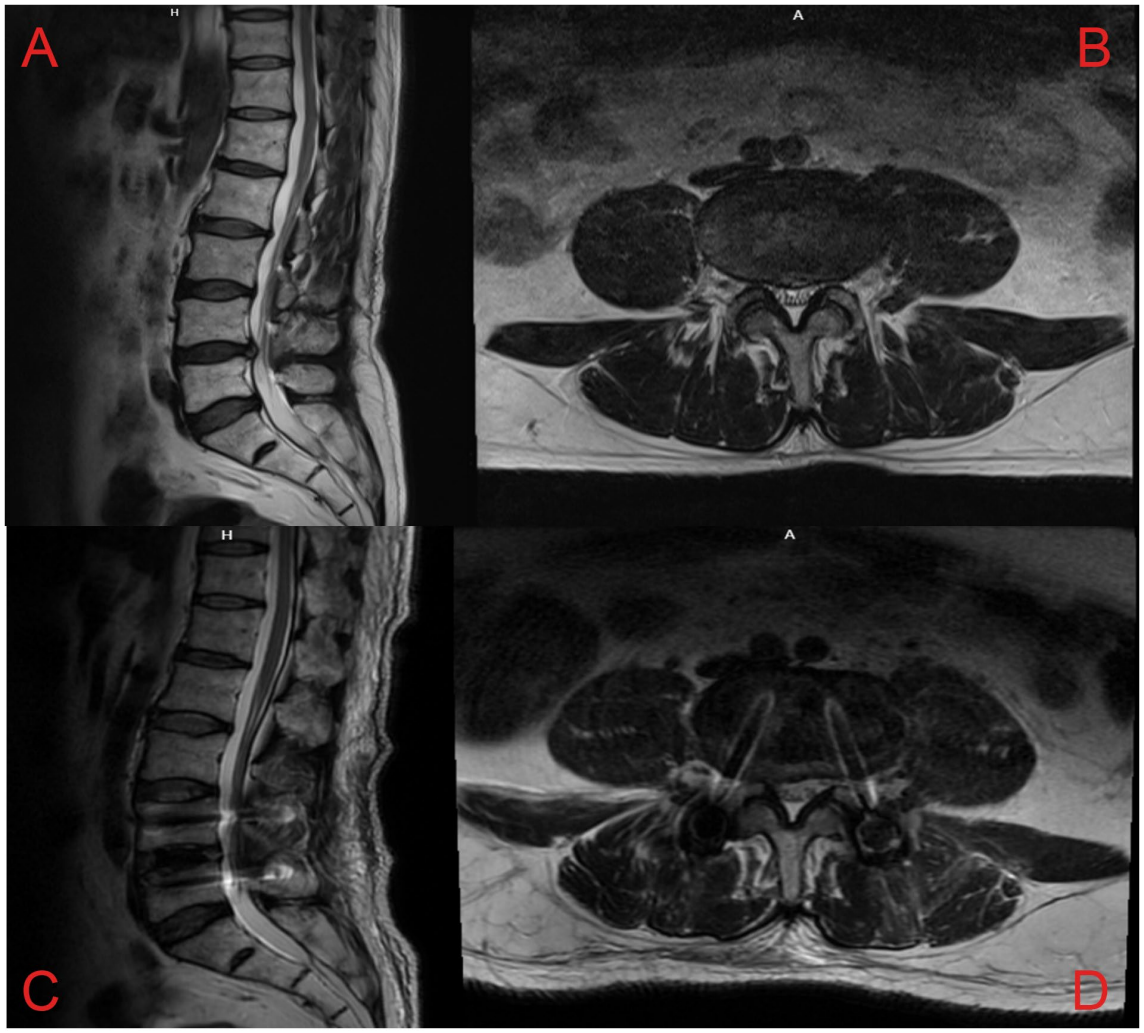
Lumbar spine MRI scans of a patient with pain and numbness in both lower extremities due to a herniated disc at L4/5. Preoperative lumbar MRI: **(A)** Sagittal T2W MRI revealed a herniated lumbar disc and decreased signal in the intervertebral disc at L4/5, **(B)** Axial T2W MRI revealed the herniated disc at L4/5. Postoperative lumbar MRI: Sagittal **(C)** and axial **(D)** images revealed the compression of the spinal cord by the intervertebral disc relieved at L4/5, and the reduction screws were fixed.

The inclusion criteria for patients were as follows: (1) Low back pain with or without lower limb pain and numbness, usually below the knee; (2) lesioned segment without paravertebral tenderness; (3) after using low-concentration lidocaine for SVN block at the intervertebral foramen, the visual analog scale (VAS) for pain was <4 points within 24 h; (4) patients had poor relief after lumbar interbody fusion (primarily involving nucleus pulposus tissue removal, interbody fusion device insertion, and titanium rod placement); (5) MRI of the lumbar spine showed decreased signal in the responsible disc and high signal at the posterior edge of the annulus fibrosus, without obvious nerve root compression; and (6) patients underwent RFA of the SVN at our department (Figure 1).

The exclusion criteria were as follows: (1) Patients with combined spinal cord tumors or myelopathy and (2) patients who had a history of postoperative lumbar or leg trauma, issues related to weight-bearing, etc.

### Data collection

Clinical information was collected from the clinical records, including age, gender, disease duration, affected side, spine level, preoperative VAS scores, VAS scores after nerve block, duration of pain relief following nerve block, and postoperative VAS scores for the lumbar region and lower extremities at 1 day, 1 month, and 3 months. In addition, preoperative, 1-month postoperative, and 3-month postoperative scores on the Pittsburgh Sleep Quality Index (PSQI) were also recorded.

The VAS assesses pain on a scale ranging from 0 to 10, where 0 indicates no pain and 10 indicates intolerable pain. Higher scores indicate greater pain intensity. The PSQI score was used to assess the patient’s sleep quality over the past 1 month. This 19-item questionnaire assesses seven aspects of sleep: subjective sleep quality, time to sleep, sleep duration, sleep disturbances, use of medications, daytime dysfunction, and subjective fatigue. Each section has four levels of scores, ranging from 0 to 3, and the cumulative score for each section is the total PSQI score, which ranges from 0 to 21. Higher scores indicate poorer sleep quality, with a total score greater than 5 suggesting poor sleep and a total score greater than 8 suggesting sleep disorders (7).

### Surgery procedures

**Figure 2:**
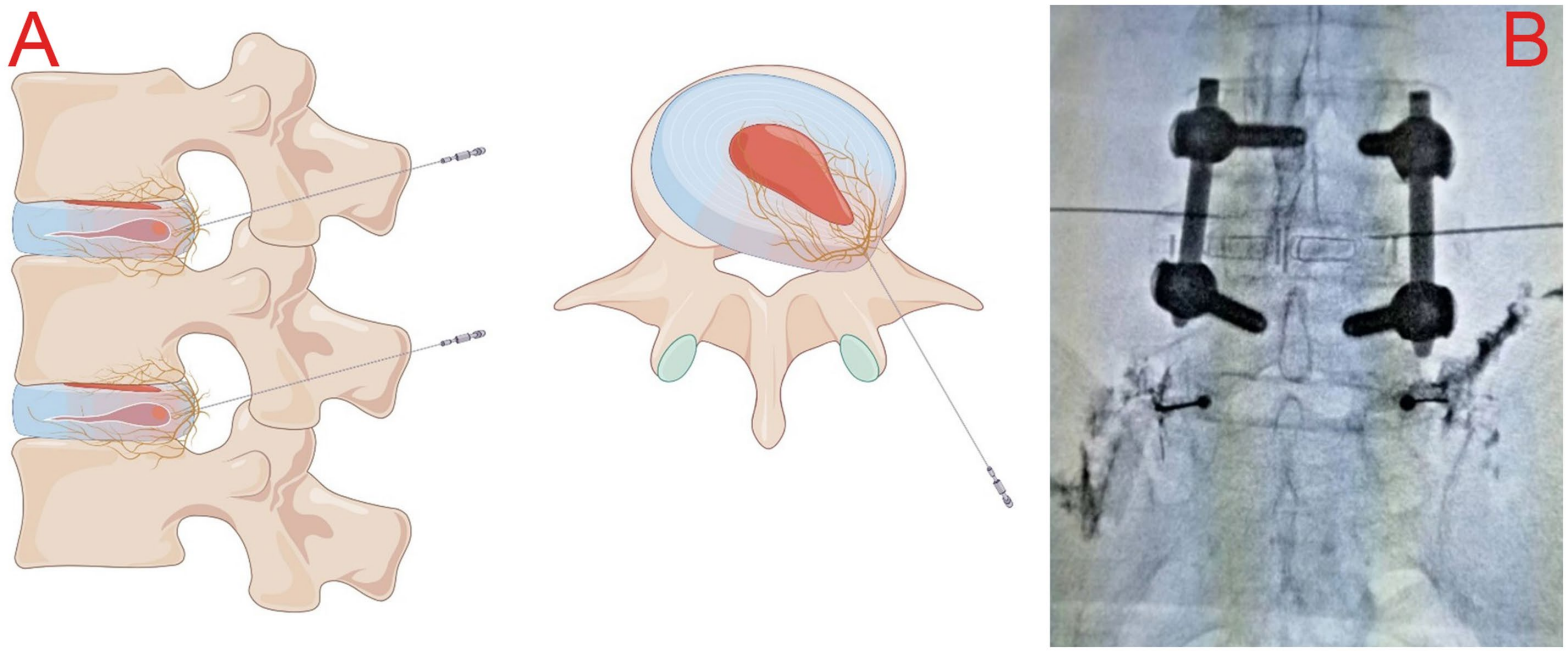
**(A)** Schematic illustration of the “safe triangle” approach for radiofrequency trocars; **(B)** intraoperative plain radiograph imaging showing the posterior interbody cage and reduction screws at the L4-L5 level, along with the appropriate location for the puncture at L4/5.

Similar to previous SVN RF-related studies (8, 9), all enrolled patients were placed in a prone position with a soft pillow under the abdomen to reduce the lumbar spine’s physiological convexity and widen the intervertebral space to facilitate needle insertion. Metal positioning markers were placed on the body surface, and the puncture route was planned under C-arm X-ray guidance, using the “safe triangle” approach on the transverse process (Figure 2A). After administering local anesthesia (3 mL of 0.5% lidocaine), a radiofrequency trocar needle (20G, Cosman Company, United States) was inserted through the intervertebral foramen to the L4-5 intervertebral space on the affected side, and a nerve block needle (20G, Camelot Company, China) was inserted into the L5-S1 intervertebral foramen. Then, 0.5 mL of iohexol contrast medium was administered to verify, under C-arm X-ray, that the contrast was located in the L4-5 intervertebral space and near the L5-S1 intervertebral foramen bilaterally (Figure 2B). The core of the puncture needle was removed, and the radiofrequency electrode was placed. A radiofrequency ablation device (G4 radiofrequency therapeutic instrument, Cosman Company, United States) was connected to perform a sensory test (50 Hz, 0.3 V), which induced pain and numbness at the site of the pain. After a motor test (2 Hz, 0.5 V) without abnormal muscle contraction in the bilateral lower limbs, radiofrequency ablation was performed at 7 Hz, 100 V, with a pulse width of 20 ms, at temperatures of 50°C, 60°C, 70°C, and 80°C. After performing thermal coagulation at 50°C, 60°C, 70°C, and 80°C for 30s each, thermal coagulation at 85°C was performed for 120 s. Then, 5 mL of an anti-inflammatory and analgesic solution (5 mL of 0.25% lidocaine, 0.5 mL of compound betamethasone, and 14.5 mL of 0.9% saline) was injected into the outlet of the L5 nerve root on the affected side via the nerve block needle to reduce local inflammatory reactions (10, 11).

After the operation, the patients were observed for 15 min, a sterile dressing was applied to the puncture point, and they were then returned to the ward. The patients received routine neurotrophic and corresponding symptomatic treatments after the operation and were discharged the following day.

### Statistical analysis

The data were analyzed using SPSS 27.0 statistical software (IBM Corp., Armonk, NY, United States). Normally distributed continuous data were presented as mean ± standard deviation (SD), non-normally distributed continuous data were presented as median (25th quartile to 75th quartile), and categorical variables were expressed as numbers (percentages). Comparisons of the continuous data were performed using a paired samples *t-*test or the Friedman test. Two-tailed *p*-values of < 0.05 were considered significant.

## Results

**Figure 3:**
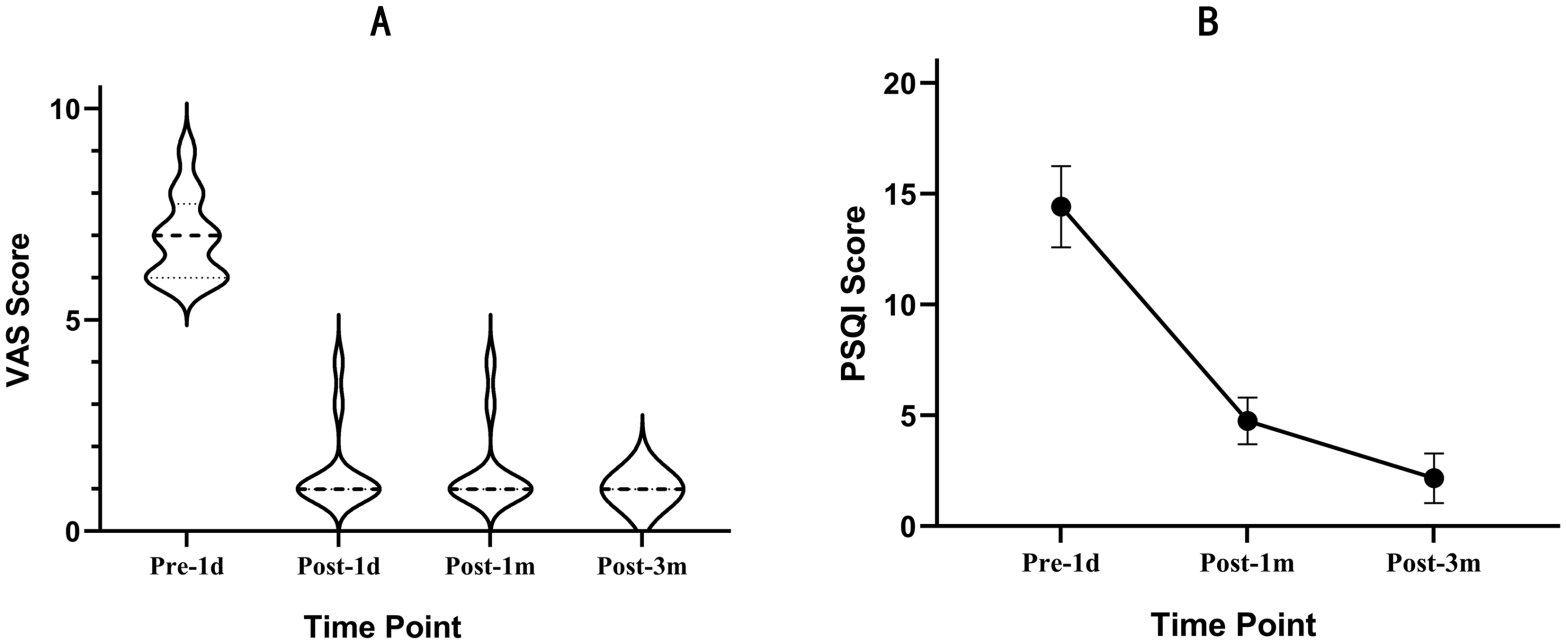
Outcomes after RFA of the SVN. **(A)** The distribution of the VAS scores at the 3-month follow-up. Data are reported as median (25th quartile to 75th quartile). **(B)** Graphs showing the perioperative changes in the PSQI. Data are reported as mean±SD.

A total of 12 patients with DLBP following lumbar fusion decompression with internal fixation on the affected side were included. All patients had undergone oblique lateral interbody fusion with internal fixation, and the complexes were retained during the RFA surgery and at the time of follow-up. The median age was 54.50 years (53.00, 61.75). The mean disease duration was 2.64 ± 2.24 years. All cases involved the L4/5 level, with six on the left side, four on the right side, and two bilateral cases (Table 1). The preoperative VAS score was 7.00 (6.00, 7.75). Using a mixture of low-concentration lidocaine and triamcinolone acetonide for intervertebral foramen responsible segmental nerve block, the VAS score decreased within 24 h compared to baseline {3.00 (2.00, 3.00) vs. 7.00 (6.00, 7.75)}. There was a significant improvement compared to before the nerve block (*p* = 0.002), and pain relief lasted for 3.92 ± 1.83 days. The postoperative VAS score at 1 day, 1 month, and 3 months was 1.00 (1.00, 1.00). This represented a statistically significant improvement compared to the preoperative period (all *p* < 0.001) (Figure 3A). The preoperative PSQI score was 14.42 ± 1.83, and the postoperative PSQI scores at 1 month and 3 months were 4.75 ± 1.06 and 2.17 ± 1.11, respectively (all *p* < 0.001) (Figure 3B).

**Table 1:** Baseline characteristics of patients with DLBP.

Characteristics	Patients (*n* = 12)
Age, years, median (25th quartile to 75th quartile)	54.50 (53.00, 61.75)
Disease duration, years, mean ± SD	2.6 ± 2.2
Sex, *n* (%)	
Male, *n* (%)	7 (58.3%)
Female, *n* (%)	5 (41.7%)
Affected side, *n* (%)	
Left, *n* (%)	6 (50%)
Right, *n* (%)	4 (33.3%)
Both, *n* (%)	2 (16.7%)
Spinal levels	
L4/5, *n* (%)	12 (100%)
Pre-operation VAS score, median (25th quartile to 75th quartile)	7.00 (6.00, 7.75)
Pre-operation PSQI score, mean ± SD	14.42 ± 1.83
VAS score after nerve block, median (25th quartile to 75th quartile)	3.00 (2.00, 3.00)
Duration of pain relief after nerve block, days, mean ± SD	3.92 ± 1.83

In addition, four cases were accompanied by local numbness in the lower limb on the painful side before operation, and the numbness was significantly relieved in two cases and slightly relieved in two cases on the first day after the operation. All patients experienced significant relief within the first month postoperatively and were completely relieved by the third month.

## Discussion

DLBP is a multifactorial degenerative disease, and its anatomical and pathophysiological mechanisms are not yet fully understood. Existing studies have shown that it may be caused by nerve irritation from the nucleus pulposus and neurogenic inflammation in the degenerated disc and that a small amount of local anesthesia administered within the disc can help relieve the pain (2, 12–14). Radiofrequency ablation of the responsible intervertebral disc inactivates the abnormally proliferating SVN branches, thereby alleviating the pain.

The intervertebral disc, which mainly consists of the nucleus pulposus and the annulus fibrosus, provides support, cushions the spine, and ensures its flexibility. Studies have found that the abnormal proliferation of the SVN and other abnormalities within the degenerated disc are the main mechanisms of DLBP (13), but the distribution of the SVN has not been fully clarified. The majority of studies have concluded that the SVN is mainly divided into superficial fibers originating from spinal nerves and deep fibers from sympathetic nerves (2, 3). The superficial nerve fibers are predominant, the deep layer is segmentally distributed, and the superficial layer is non-segmental. In addition, the SVN distributed in adjacent segments may intersect (15–17). Degeneration of the intervertebral disc includes dehydration, loss of height, abnormal proliferation of the SVN, and rupture of the annulus fibrosus (14). Anatomical findings show that normal intervertebral discs are poorly innervated, with nerve fibers distributed in the annulus fibrosus and endplate, gradually degenerating toward the outside of the annulus fibrosus with age. However, when the intervertebral disc undergoes degeneration and other pathological changes, pro-inflammatory factors can stimulate the abnormal proliferation of nerves within the intervertebral disc and induce the growth of the SVN toward the nucleus pulposus (12, 18–21). Inflammatory factors within the annulus fibrosus can stimulate adjacent nerves, causing lower extremity pain in the innervated area, without significant nerve compression on imaging (9, 17). In addition, basic findings suggest that inflammatory stimulation is more important than mechanical compression because it lowers the threshold of nerve excitation and causes changes in neurophysiology, ultimately resulting in pain (13).

Nucleus pulposus removal combined with interbody fusion relieves nerve irritation and decompression (22). The procedure mainly involves removing the degenerative intradiscal nucleus pulposus tissue at the responsible segment, inserting the interbody fusion device and autologous bone, and stabilizing the area with titanium rods (23). Intraoperative retention of a portion of the annulus fibrosus helps maintain the stability of the fused segment and contributes to partial weight bearing and stress dispersion. Clinically, partial preservation of the annulus fibrosus is usually considered for spinal stability, but it can also lead to chronic pain due to the activation of the abnormally proliferating SVN within the annulus fibrosus (24, 25). Therefore, surgery may be less effective for DLBP that is not caused by nerve root compression. In this study, all patients underwent oblique lateral interbody fusion with internal fixation; however, they presented to our department with poor outcomes and denied any history of postoperative trauma. For patients experiencing pain in whom disc compression has been ruled out, nerves in the intervertebral disc, such as the SNV, become a major consideration. RFA has been widely used in the treatment of various types of neuropathic pain. In addition, in DLBP, RFA inactivates the neoplastic nerves in the intervertebral discs and denatures the adjacent protein matrix, reducing the inflammatory stimulus and allowing the discs to be partially retracted and stabilized (26, 27).

At present, the mechanism of DLBP is complex; however, as research advances on intradiscal mechanisms such as the SVN, corresponding therapeutic concepts and measures continue to evolve. In the above case series, after the limited effectiveness of nucleus pulposus removal with internal fixation, RFA was used to target the abnormally proliferated SVN and the inflammatory substances within the disc, relieving the pain and further confirming the pathological mechanism of SVN-mediated DLBP. In addition, lumbar fusion was performed to alleviate the long-term compression of the nerve roots, and the steroids administered after RFA further promoted the repair of the nerve roots. This may explain the gradual improvement of symptoms in the four patients with concomitant lower extremity numbness.

There are some limitations to this study. First, this was a single-center case series study of patients with DLPB who underwent RFA following lumbar spine surgery. The small number of cases and the short follow-up period should be considered when interpreting our results. Second, since this study was retrospective, we did not apply discography, which has some risks and is not routinely used in clinical practice. Instead, we used a transforaminal block of the SVN to assist in the diagnosis. Discography will be used as a diagnostic criterion in future prospective studies. Third, although steroids combined with radiofrequency ablation are a common approach for treating neuropathic pain, resulting in a persistent decline in the VAS and PSQI scores as expected, the follow-up period still needs to be extended to verify the long-term effectiveness of RFA treatment and further explore the reasons behind its clinical efficacy.

## Conclusion

Radiofrequency ablation of the SVN provides satisfactory short-term clinical results in patients with DLBP following lumbar interbody fusion. This appears to be an effective treatment for patients with DLBP who have poor outcomes after open lumbar spine surgery.

## Data Availability

The datasets presented in this article are not readily available because the datasets used and/or analysed in the current study are available from the author upon reasonable request. Requests to access the datasets should be directed to Lijie Wang, wanglijie07001558@163.com.
